# Polycystic ovary syndrome: emerging stem cell therapies

**DOI:** 10.1590/1806-9282.20231436

**Published:** 2024-07-19

**Authors:** Karimat Adeola Busari, Pinar Tulay

**Affiliations:** 1 Near East University, Faculty of Medicine, Department of Medical Genetics – Nicosia, Cyprus.; 2 Near East University, DESAM Research Institute – Nicosia, Cyprus.; 3 Near East University, Center of Excellence, Genetics and Cancer Diagnosis-Research Center – Nicosia, Cyprus.

## INTRODUCTION

Polycystic ovary syndrome (PCOS) is a highly prevalent endocrine disorder that affects females aged 15 to 49 years, with a frequency of up to 20%^1^. PCOS is a heterogeneous condition and is characterized by chronic anovulation and hyperandrogenism. In addition to being linked to metabolic abnormalities, PCOS raises the risk of developing type 2 diabetes, cardiovascular disease (CVD), and endometrial cancer. Insulin resistance (IR) is thought to be the cause of the metabolic abnormalities associated with PCOS and the inefficient metabolism of carbohydrates, which raises the risk of CVD. PCOS is a major threat to public health because of its metabolic, reproductive, and psychological features. It has been diagnosed in more than 105 million females aged 15–49 years globally. PCOS can cause a variety of symptoms, which include abnormal menstrual periods, infertility, enormous growth of the hair, and issues with pregnancy. Furthermore, PCOS is linked to psychological disorders such as depression, anxiety, and also low self-esteem. As age increases, the condition advances from reproductive illness to a metabolic disorder^1-3^.

Obesity (BMI ≥30 kg/m^2^) and overweight (BMI 25–29.9 kg/m^2^) are risk factors linked to PCOS that have been identified as significant contributors to global health issues in women. Obesity has also been linked to reproductive health issues such as anovulation. The incidence of anovulation increases substantially with body weight. Gaining abdominal fat indicates a higher risk of IR, which is another factor contributing to difficulties with reproduction. Increased testosterone production and anovulation have been linked to IR in obese women^4^. It has been proposed that bisphenol A is an endocrine disruptor that mimics estrogen. BPA is an exogenous substance that is regarded as a xenoestrogen because it imitates the effects of 17-β estradiol. As a result, BPA can interfere with E2 feedback at the ovarian and hypothalamus-pituitary levels, which inhibits the actions of the HPO axis. BPA can also lead to fertility problems, irregular menstruation cycles, insufficient release of hormones, and problems with the growth and performance of the female reproductive system^5^. Researchers discovered that the effect of progesterone may be hampered by hormonal (elevated testosterone levels) and metabolic (elevated insulin levels) abnormalities. In PCOS, progesterone therapy does not completely resolve the histomorphometric abnormalities of the endometrium, which are associated with insulin and androgen levels^6^.

Due to the complex causes of PCOS, treatment is typically customized based on the patient's current signs and symptoms rather than being mono-therapeutic. Complementary therapies have been recommended for PCOS treatment and management. The cornerstone of PCOS management is thought to be modifications to diet and lifestyle. Current treatments typically improve symptoms or consequences related to the conditions, such as menstrual disruption, elevated testosterone levels, fertility issues, metabolic abnormalities, and cancer and CVD prevention, without tackling the underlying cause. While ovulation stimulation works, rates of pregnancies are low, necessitating the use of advanced assisted reproductive procedures^7^.

The endometrial morphological and genetic alterations may have a major role in PCOS women's fertility. As PCOS is frequently linked to IR, obesity, and metabolic abnormalities, the ability of melatonin to enhance metabolic health is crucial for those who have the illness. As melatonin lowers oxidative stress and increases insulin sensitivity, it provides an all-encompassing strategy for treating the metabolic factors that cause PCOS. Increased sensitivity to insulin can lessen the effects of hyperinsulinemia and the hazards it carries, including diabetes and cardiovascular problems^8^. In the rat model of PCOS (permanent estrus), melatonin seems to restore the regular process of granulosa cell proliferation, according to a study. Melatonin therapy is a low-toxicity, readily available, reliable option. Based on the research, melatonin is beneficial for treating and preventing reproductive issues in PCOS patients^9^.

In terms of PCOS-specific measures (LH: FSH ratio, serum testosterone, and serum AMH), women with PCOS benefit from a micronutrient supplementation, administered for a minimum of 3 months, which contains omega-3 fatty acids, catechin, folic acid, vitamin E, glycyrrhizin, selenium, and co-enzyme Q10^10^. Following the induction of PCOS in rats, the treatment of clomiphene citrate enhanced the structure and physiological functions of rat ovaries^11^. Co-enzyme Q10 (CoQ10) and clomiphene citrate therapy together significantly increase ovulation rate in PCOS-affected women in a way that is not possible with clomiphene citrate therapy alone. An adjuvant for clomiphene citrate that may be effective is CoQ10. Before attempting a more challenging treatment like gonadotrophins or laparoscopic ovarian drilling, a combination of CoQ10 and clomiphene citrate has been demonstrated to be an effective, economical, and safer way to increase follicular growth in PCOS patients^12^.

## REGENERATIVE MEDICINE: STEM CELL THERAPY

The primary goal of treatment, such as pharmaceutical therapies, is to alleviate symptoms. In addition, some of the patient's issues can be treated and their severity can be decreased by surgical procedures such as bariatric surgery and assisted reproductive technologies. In general, utilizing pharmaceuticals over a lengthy period elevates the probability of side effects. In addition, surgical approaches can result in risky outcomes. Here, it is clear that PCOS requires a novel, interdisciplinary approach. Stem cells can be used in regenerative medicine to create an effective alternate strategy for treating PCOS. Animal models can also help us understand PCOS's genetic changes and disturbed metabolic pathways. In preliminary clinical stages, they can also be used to test new treatments^13^.

### Induced pluripotent stem cells

Present research in cell reprogramming employs iPSC derived from patient somatic cells to generate stem cells specific to the patient for studying the disease etiology and generating specific treatments. iPSCs have been used in disease models to know the causes and also test potential treatments. When developed into appropriate cell types, the majority of iPSCs exhibit detectable characteristics particular to the disease. iPSC lines were created from the somatic cells of PCOS patients exhibiting the common symptoms. RNA microarray was used to examine the transcriptional patterns of these iPSC lines, as well as metabolic capabilities and mitochondrial activity in the iPSCs derived from patients in vitro. Following that, metformin was utilized to show response in the iPSCs model derived from the patients to assess the possible benefit of the iPSCs for drug development and future clinical applications. The derived iPSCs provide a novel biological cell framework for studying PCOS pathophysiology and aiding in the discovery of new medications for clinical applications^14^.

### Mesenchymal stem cells

Mesenchymal stem cells have a homing ability, where they release a variety of growth factors, cytokines, and extracellular vesicles with antifibrotic, immunosuppressive, antiapoptotic, anti-inflammatory, and angiogenic properties. MSCs are gaining popularity as a promising cell-based therapy because they offer various benefits over other sources, such as more accessibility, less ethical issues, and a great capacity for self-renewal and differentiation. MSCs have been studied therapeutically in female infertility by medical researchers. In particular, several research studies have concentrated on MSCs as a method for recovering ovarian function and infertility treatment. MSCs can be obtained from dental pulp, umbilical cord tissue, bone marrow, amniotic fluid, adipose tissue, placental tissue, Wharton jelly, and pluripotent stem cells, among other places. MSCs have therapeutic significance through the capability to divide into diverse cell lineages and influence immune responses through the modulation of the immune system. MSCs have been found to reactivate endometrial function and enhance the outcomes of pregnancy^15-17^.

Mesenchymal stem cells express their influence through modulating several molecular and biological processes. miRNAs, particularly exosomal miRNAs, seem to have a major role in modulating MSC impacts and hence represent potential targets for therapy^18^. Studies have enhanced our knowledge of the methods and treatment opportunities of stem cell therapies for gynecologic conditions that affect the function of the reproductive tissue. These studies have paved the way for developing innovative and efficient MSC-based therapies, which have the possibility of helping women suffering from ovarian insufficiency or infertility through the restoration of their reproductive health and enhancing their life quality. Additional study is needed to know the safety and effectiveness of MSC in the treatment of infertility^19^. PCOS patients have elevated levels of expression of the *CYP17A1* and *CYP11A1* genes, which are implicated in androgen synthesis. The efficiency of human MSCs (hMSCs) was investigated as a potential cell therapy through the injection of hMSCs into the ovaries of a mouse model. It demonstrated a substantial decrease in hyperandrogenemia, and hMSCs release a variety of factors in the media, which is referred to as the secretome. The secretome of hMSCs comprises factors that can suppress androgen production in theca cells. MSCs release proteins that decrease androgen production through the inhibition of steroidogenesis proteins *CYP11A1* and *CYP17A1* gene expression^20,21^.

### Umbilical cord mesenchymal stem cells

Umbilical cord mesenchymal stem cells are considered to have an unlimited supply of stem cells, and they represent an intriguing stem cell source for regenerative medicine. They can be extracted in vast quantities from human umbilical cords and grown in cultures. It was discovered that after administering UCMSCs, the endocrine function is restored in the synthesis of estrogen with an increase in the weight of estrogen-dependent organs, such as ovaries, and the exocrine function is also restored resulting in successful pregnancies and delivery of the healthy children. Many other researchers revealed similar findings^22^.

New research shows that UCMSC transplantation may be useful in improving PCOS pathological changes. It was discovered that transplantation of UCMSC improved ovarian function in mice with DHEA-induced PCOS^21^. This impact was interceded by a decrease in the synthesis of proinflammatory cytokines, such as interleukin 1 beta and tumor necrosis factor-alpha, and fibrosis-associated genes, such as connective tissue growth factor, while increasing the secretion of anti-inflammatory factors like IL-10 in local ovarian and uterine tissues, implying that UCMSCs may change a proinflammatory condition to an anti-inflammatory condition. The finding of this research was that PCOS was alleviated in the mice by stopping inflammation. While these results imply that UCMSC transplantation may be useful for managing ovarian diseases, additional research is needed to create a reliable USMSC-based therapy method^23,24^.

### Bone marrow mesenchymal stem cells

The transplantation of BM-MSCs significantly enhances folliculogenesis in PCOS-induced mice. Stem cells produced from bone marrow are widely employed for a variety of therapeutic applications, but clinical use for the treatment of female infertility has remained difficult to find so far^16^. Despite this, a recent study using mouse models of PCOS demonstrated improvements in the function of the endocrine, quality of the oocytes, and folliculogenesis due to PCOS's anti-inflammatory, anti-apoptotic, and anti-oxidative qualities^16^. Another study found that after intra-ovarian delivery of BM-MSCs, older women (who had previously been subjected to chemotherapy) had important recovery of ovarian function. The number of oocytes grew enormously as folliculogenesis developed; however, the quantity of primary and pre-antral follicles decreased substantially.

Chugh et al. found out that BM-hMSC and its secreted components (secretome) demonstrate powerful anti-inflammatory properties. In both in vitro and in vivo models of PCOS, the therapeutic effectiveness of BM-hMSC and its secretome was assessed. The effectiveness of intra-ovarian injection of BM-hMSC in the treatment of phenotypes associated with PCOS such as metabolic and reproductive abnormalities was demonstrated. This method might be a potential treatment for PCOS patients. Our findings demonstrate that BM-hMSC can inhibit inflammation induced by PCOS via IL-10 production. The anti-inflammatory cytokine IL-10 performed an important function in relaying BM-hMSC effects. BM-hMSC might be an effective therapeutic option for PCOS^25,26^.

Increased inflammation and the synthesis of androgen from ovarian theca cells are PCOS primary characteristics, and approximately 50% of women with PCOS have elevated production of androgen. Therefore, H295R (androgen-producing human adrenocortical-carcinoma cells) serves as an ideal in vitro PCOS model. The effects of H295R cells exposure to the BM-hMSC secretome and IL-10 were studied. Comparable experiments were conducted on primary cultured theca cells derived from PCOS patients. In both H295R cells and primary cultured theca cells, the BM-hMSC secretome inhibited the production of steroids, inflammation, and androgen synthesis^25,27^.

## CHALLENGES AND FUTURE PERSPECTIVE

One of the many unanswered questions about PCOS is regarding the common factor that connects obesity, hyperandrogenism, and IR. Proper diagnosis and treatments are required for women who may develop infertility during their years of reproduction in order to enhance their prognosis. Although PCOS is manageable with lifestyle modifications and medications, a majority of women with PCOS do not receive proper treatment due to the low rate of accurate diagnosis of this disorder^28-30^.

The transplantation of stem cells or their derivatives into the relevant tissues or organs is one of the most potential treatments for many fatal conditions. However, because of the sensitivity and complex nature of the immune system, obtaining immune-competent cells from any patient is challenging. In this context, iPSCs and technologies for gene editing may be useful in generating functional autologous cells. However, with major technological breakthroughs in reprogramming, iPSCs are not yet suitable for transplantation into patients, except for a few in current clinical research. There have been few publications on the equivalent of the molecular background and functions of human ESCs and iPSCs, and the iPSCs genome and epi-genomic integrity must be thoroughly assessed before they can be used in treatments^31^. Also, when contemplating stem cell treatment, the first issue for the researchers is to unravel the processes behind the way stem cells act in the damaged microenvironment using animal models and then translate the outcomes of these studies to humans. The second issue is to detect and extract stem cells from the tissue before inducing differentiation into the appropriate cell types. The final issue is to prevent immune rejection following the transplantation of stem cells. Immune rejection is a significant impediment to effective stem cell transplantation^32^.

## DISCUSSION

There is optimism about developing an effective therapy method based on the regenerative features of stem cells for reproductive issues. However, to achieve major developments for the treatment strategy, an accurate and effective study plan, from the isolation of stem cells to informed voluntary consent and cell delivery model, is crucial for the success of initial clinical trials considering previously published studies to overcome the limitations. There is no remedy or cure because therapies have so far focused on the symptoms rather than the syndrome itself. A comprehensive attempt should be made to completely research the condition so as to enhance the treatment and hinder the devastating long-lasting results of this condition on the well-being of patients. For the early diagnosis and screening of PCOS subtypes, crucial genetic polymorphisms may be effective. Finding efficient preventative tactics and therapy modalities will require additional research on the genetics and pathophysiology of PCOS. Prebiotic, probiotic, and synbiotic administration seems to enhance a number of biochemical outcomes and has positive benefits on women with PCOS, while the underlying mechanisms are yet unclear. The importance of these drugs in PCOS therapy or perhaps prevention requires further study.

## CONCLUSION

Stem cell studies have brought about major advancements in the treatments of reproductive issues. Still, further pre-clinical and clinical trials are needed to establish the advantages of different stem cell therapies and discover the best stem cell type or source for subsequent studies. According to numerous studies, PCOS-related obesity and metabolic syndrome can be treated with novel or modified medications. Statins, letrozole, and nutrient supplements have been the subjects of recent research studies for the treatment of PCOS. To demonstrate the efficacy of novel and emerging treatments, such as aromatase inhibitors and IL-22 therapy, as well as others, in effectively managing PCOS, more research is needed.
